# Antioxidant Lifestyle, Co-Morbidities and Quality of Life Empowerment Concerning Liver Fibrosis

**DOI:** 10.3390/antiox9111125

**Published:** 2020-11-13

**Authors:** Diego Martinez-Urbistondo, Rafael Suarez del Villar, Josepmaria Argemí, Lidia Daimiel, Omar Ramos-López, Rodrigo San-Cristobal, Paula Villares, Jose Alfredo Martinez

**Affiliations:** 1 Internal Medicine Department, Hospital HM Sanchinarro, HM Hospitales, 28050 Madrid, Spain; rafasdvc@gmail.com (R.S.d.V.); pvillares@hmhospitales.com (P.V.); 2 Liver Unit, Clínica Universidad de Navarra, Centro de Investigación Médica Aplicada, 31008 Pamplona, Spain; jargemi@unav.es; 3 Precision Nutrition Program, Instituto Madrileño de Estudios Avanzados, Universidad Autónoma de Madrid, 28049 Madrid, Spain; lidia.daimiel@imdea.org (L.D.); rodrigo.sancristobal@imdea.org (R.S.-C.); jalfredo.martinez@imdea.org (J.A.M.); 4 Medicine and Psychology School, Autonomous University of Baja California, Tijuana 22390, Mexico; os_mar6@hotmail.com; 5 CIBERobn: Fisiopatología de la Obesidad y Nutrición, Instituto Carlos III, 28029 Madrid, Spain

**Keywords:** antioxidant lifestyle, comorbidity, quality of life, liver fibrosis, health empowerment, wellbeing, redox status

## Abstract

The assessment of liver fibrosis has gained importance since the progression of non-alcoholic fatty liver disease (NAFLD). Indeed, the description of the association between undetected liver fibrosis and lifestyle in terms of antioxidant habits, comorbidity and quality of life (QoL) domains may help in the characterization of subjects with NAFLD. A cross-sectional evaluation of (*n* = 116) consecutive patients from an Internal Medicine ambulatory evaluation was performed. Demographic data, lifestyle, co-morbidity, QoL (according to the SF-36 index) and analytical values to calculate the oxidative related Fibrosis-4 (FIB-4) index were recorded. The association between FIB-4 and co-morbidity, antioxidant habits in QoL was assessed in univariate analysis (*p* < 0.05) and confirmed in multivariable analysis for 4 of the 8 SF-36 categories: Physical QoL, Physical role, Social QoL and General QoL, as well as in the Physical summary of SF-36 (*p* < 0.05). Finally, interactions were assessed between co-morbidity, FIB-4 and antioxidant habits showed in the prediction of mean SF-36 (*p* < 0.01). Liver fibrosis assessed by the oxidative surrogate index FIB-4 is associated with the interaction between antioxidant lifestyle, co-morbidity and physical, social and general aspects of QoL in apparent liver disease-free individuals, generating a proof of concept for health empowerment and personalized medicine.

## 1. Introduction

Liver fibrosis is the result of an oxidative hepatic pathological condition involving inflammation, lipotoxicity, cell infiltration and unbalanced redox processes, which may lead to liver function impairment accompanying hepatocyte damage [1]. Liver fibrosis screening has been traditionally focused on patients at risk, due to the noticeable prevalence of the disease in defined subgroups of the population, such as Hepatitis B Virus- and Hepatitis C virus-infected subjects, alcohol abusers and autoimmune or deposit disease patients [2]. Nevertheless, the epidemiology of liver disease has suffered a profound evolution in most developed countries [3], with the progression rates of non-alcoholic fatty liver disease (NAFLD). This disease differs from other hepatic dysfunctions in the apparent absence of a unique trigger to explain NAFLD onset [4]. Alongside this, this disease is more challenging than other liver disorders due to the large and heterogeneous population at risk. Thus, the detection and stratification of patients with NAFLD needs a broad analysis of the general population. Two factors contribute to the complexity in evaluating NAFLD in a clinical setting: the lack of a reliable non-invasive gold standard for the diagnosis, and the multi-causal association in the origin of this pathology [5].

Liver biopsy is still the reference standard for NAFLD monitoring [6]. Nevertheless, the absence of reliable non-invasive follow-up tools and precision therapeutic measures in patients at early stages of this disease discourages the routine performance of invasive procedures in a clinical real-life setting [7]. For this reason, different objective markers have been devised for the detection and follow-up of liver fibrosis [8]. These methods are of special interest in NAFLD due to the large population at risk. Among them, the oxidative proxy Fibrosis-4 index (FIB-4) has demonstrated prognostic accuracy, longitudinal sensitivity and a linear value in patients with different chronic liver diseases, including NAFLD [9,10,11]. In addition, FIB-4 assessment is cheap, reproducible and easy to interpret, becoming a very suitable index in this scenario [12]. Additionally, FIB-4 has been associated with oxidative stress, which is one of the main causes of fibrosis in NAFLD and might be used as indirect marker of oxidative balance in NAFLD-suspected patients [13].

NAFLD emerges as a multidimensional network of interactions [14]. Lifestyle is a core aspect in fatty liver development and liver fibrosis since several dietary and exercise patterns have been described to reduce or boost the development of this disease [15]. Antioxidant status is one recognized approach to comprehending NAFLD, due to the impact of the redox balance in the different pathways of NAFLD progression, including insulin resistance, chronic inflammation and oxidative stress [16,17]. Although the implementation of an anti-oxidative lifestyle in patients may seem difficult, there are specific risk factors, which can be overcome while being practical in a medical setting. In this context, simple measures such as the avoidance of dietary ultra-processed products and carbonate beverages have an impact on the NAFLD outcome while promoting healthy dietary intakes [18]. In the same way, avoiding sedentarism might play a role in NAFLD prevention and correction. Indeed, a reduction in quality of life (QoL) has been reported in patients with NAFLD, so it was concluded that physical activity approaches to improving QoL may be implemented in patients at liver disease risk [19]. Chronic non-communicable diseases are also associated with fatty liver development [20]. The increase in the disease burden due to lifestyle and ageing should also be taken into account when evaluating liver fibrosis due to fatty liver infiltration, where oxidative patterns may be involved [21]. Indeed, the redox balance in the body depends on external oxidative and antioxidative inputs, including specific nutrients and physical activity patterns as well as on homeostatic processes and reactive oxygen species (ROS) production [22].

Finally, scientific efforts are driven to the change from the raw analysis of survival to a “precision medicine” approach [23]. In this context, quality of life (QoL) plays a key role in the evaluation and treatment of the diseased, with a potential impact on lifestyle practices and dietary intake, which has been scarcely investigated. [24]. The growing importance of QoL with potential impact on lifestyle practices and dietary intake in patients evaluation has led to the development of different tools in the objective assessment of this health aspect [25]. In this context, the SF-36 index is a concise resume of QoL, with reliable results in both investigation and clinical practice to measure physical and mental wellbeing [26]. Indeed, QoL and NAFLD have been associated through co-morbidity and lifestyle [27]. Interestingly, QoL objective assessment could help in the understanding of NAFLD detection and development, as well as the endogenous relationship with the oxidative status at the cell level.

The objective of this study is to evaluate the association of FIB-4 index score, a non-invasive liver fibrosis scale, with lifestyle in terms of antioxidant habits, co-morbidity and quality of life in liver disease free patients in a clinical setting to set a proof of principal of the linkage between undetected oxidative liver fibrosis, metabolic status and lifestyle

## 2. Materials and Methods

### 2.1. Population

The study involved 116 consecutive patients, who to an internal medicine ambulatory evaluation in a Spanish tertiary hospital between October 2018 and March 2019 and filled a validated lifestyle questionnaire [28] as well as the SF-36 v2 form [26] and provided sufficient analytical results to calculate FIB-4 index (inclusion criterion)and declared no prior liver disease (exclusion criterion). The attending physician collected morbidities included in the Charlson Comorbidity Index [29]. The laboratory tests were ordered independently from the study evaluation. This research was approved by the Center Bioethics Board (ESCAVIDA/04) and followed the Helsinki Good Practice commitments and legal requirements in Spain.

### 2.2. Variables and Co-Variables

Fibrosis—4 index was calculated according to the original formula [9]:

(Age × GOT)/(Platelet count (mm^3^) × (GPT^1/2))

This variable was the measurement used to evaluate study processes, which were liver oxidative status in terms of fibrosis. The eight domains of the SF-36 and the physical SF-36 summary were calculated applying previously validated formulas [26]. Oxidant lifestyle was considered as the addition of dietary habits and exercise. Considering the dietary aspects of lifestyle, pro-oxidative habits were considered when patients declared a consumption of ultra-processed pastries more than three times a week or carbonated drinks more than once daily. Pro-oxidative physical activity was considered when patients declared no exercise performance in their free time, at least once a week based on the NW index [28]. Pro-oxidative lifestyle was considered as the sum of one point if the patient declared any of the pastries or carbonated beverages consumption, while another point was added if the patient declared no regular physical activity, to a range of 0–2 points (Appendix A). Co-morbidities were expressed as the simple sum of the co-morbidities recorded in the Charlson Comorbidity Index as described elsewhere [29].

### 2.3. Statystical Analysis

Chi-square test was applied to qualitative variables while T-student and one-way ANOVA tests were applied to binomial and multinomial analysis of quantitative variables in the univariate analysis. Multivariable regression models were developed to predict FIB-4 with variables found to be *p* < 0.05 in the univariable analysis. Factorial ANOVA analysis were performed to evaluate effect modification between FIB-4, co-morbidity, SF-36 categories and oxidative lifestyle. Multivariable analyses were performed, including morbidity due to the impact of disease in liver oxidative stress and pro-oxidant habits and QoL domains to prove their association with FIB-4 in the individuals studied. To avoid co-linearity, age was not included in the multivariable models due to the presence of age in the FIB-4 equation. Results were considered statistically significant with a *p* value < 0.05. The IBM SPSS statistical package v20.0 (Chicago, 2018) was used to perform the analysis, whose manual was followed.

## 3. Results

The study population presented a mean age of 58.5 ± 18.1 years. Female participants constituted 59% of the sample. The disease burden of the population accounted for 0.93 co-morbidities per patient. Frequent medications were recorded (Table 1). No supplements or nutraceuticals were declared by the patients in the present population. About 49% of the patients of the study population had at least one dietary oxidant habit, and 20.7% of the patients did not perform any exercise. Hepatic liver fibrosis was measured by the surrogate FIB-4 index, with a mean of 0.65. The mean for the 8 categories of QoL as recorded in SF-36 was 78 ± 28 points for the physical QoL, 76 ± 25 for the physical role, 86 ± 22 for the emotional role, 70 ± 20 for the mental QoL, 79 ± 26 for the social QoL, 55 ± 23 in vitality, 64 ± 27 in pain assessment and 55 ± 20 in general health. The SF-36 physical summary scored 63 ± 23 points (Table 1).

In the univariate analysis, first an analysis of the association between FIB-4 components (age, GOT/GPT ratio and platelets) was performed (Appendix A Table A2). After the confirmation of the additive capacity of each component to the prediction of QoL categories, FIB-4 was analyzed through the different population characteristics. This index was associated to an increase in the disease burden (no disease 0.51, a single disease 0.75, more than one disease 1.01; *p* < 0.001). The adherence to a pro-oxidant lifestyle was also associated with a higher FIB-4 score (no pro-oxidant habits 0.57, one pro-oxidant habit 0.77, two pro-oxidant habits 0.99; *p* < 0.04). Concerning the quality of life assessment, four categories were found to be associated with a lower FIB-4: physical category (Tercile 1: 0.90, Tercile 2: 0.76, Tercile 3: 0.44; *p* = 0.002), physical role category (Tercile 1: 0.89, Tercile 2: 0.55, Tercile 3: 0.61; *p* = 0.027), social category (Tercile 1: 0.94, Tercile 2: 0.47, Tercile 3: 0.63; *p* = 0.005), general health (Tercile 1: 0.89, Tercile 2: 0.64, Tercile 3: 0.50; *p* = 0.016) and physical summary categories of SF-36 (Tercile 1: 0.89, Tercile 2: 0.58, Tercile 3: 0.51; *p* = 0.012) as shown (Table 2).

Several multivariable analyses were performed to predict FIB-4 as a dependent variable, including sex, morbidity, pro-oxidant lifestyle and each of the analyzed SF-36 categories (Table 3), which revealed that morbidity remained independently associated with FIB-4 in all the models, while antioxidant lifestyle was found *p* < 0.05 in the physical role, social and general health categories. Additionally, every QoL category was independently associated with FIB-4 in their relative models.

**Figure 1:**
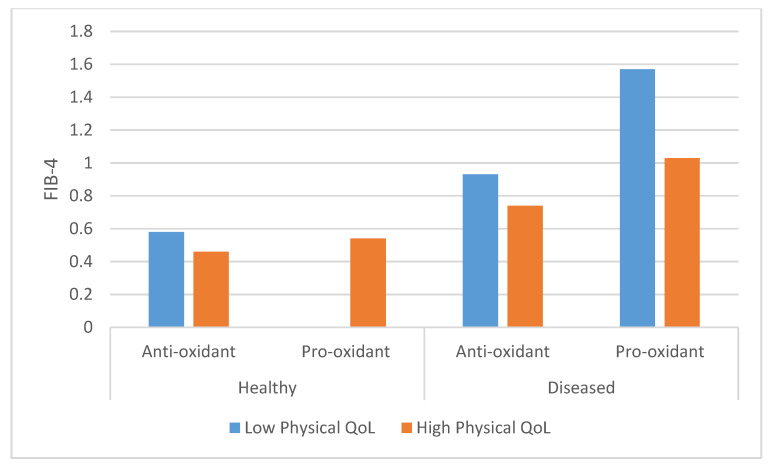
Fibrosis-4 (FIB-4) according to comorbidity, oxidative lifestyle and physical summary quality of life (QoL) SF-36. Antioxidant: Patients with 2 points in pro-oxidant lifestyle vs. rest of the population; Healthy: No diseases; Comorbid: At least 1 disease; Low physical QoL: Lowest tercile of SF-36 Physical summary. Statistical analysis: Comorbidity *p* = 0.01; Oxidative lifestyle *p* = 0.04; SF- 36 Physical Summary QoL *p* = 0.05.

Furthermore, the results of the multivariable analysis of the physical summary of the SF-36 questionnaire evidenced the independent, statistically significant association of co-morbidity, oxidant lifestyle and physical QoL with the FIB-4 index results (Figure 1).

**Figure 2:**
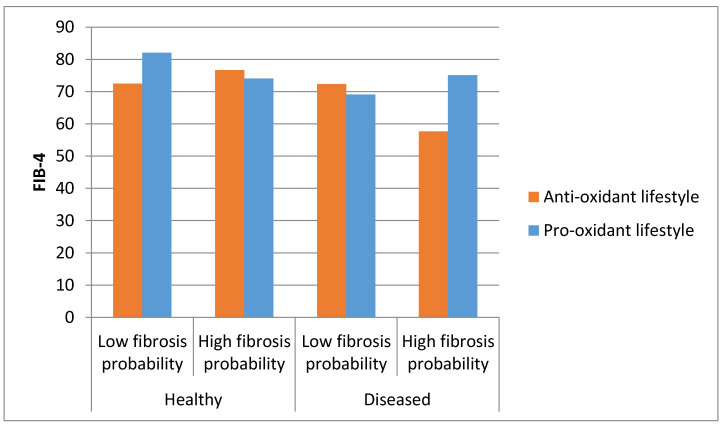
Interaction between comorbidity, liver fibrosis probability according to FIB-4 and oxidative lifestyle in the prediction of QoL. QoL assessed by the mean of all 8 categories in SF-36; Low fibrosis probability: *p* < 50 of FIB-4; High fibrosis probability: *p* > 50 of FIB-4; Anti-oxidant lifestyle: 0 or 1 oxidative factors; Pro-oxidant lifestyle: 2 oxidative factors; Diseased: 1 or more co-morbidities. *p* for interaction between comorbidity, liver fibrosis and oxidative lifestyle < 0.001.

Finally, a new model was designed to evaluate the interaction between comorbidity, liver fibrosis probability according to FIB-4 and oxidative lifestyle in the prediction of QoL, using the mean of the SF-36 score as QoL surrogate. The results (Figure 2) show that the interaction between co-morbidity and fibrosis has an influence on the effect of an antioxidant lifestyle on quality of life. In this context, an antioxidant lifestyle would be associated with a better quality of life in patients with either more liver fibrosis or co-morbidity, while a pro-oxidant lifestyle would be related to a better quality of life in healthy patients with less liver fibrosis and in diseased patients with liver fibrosis. The interaction between co-morbidity, fibrosis and lifestyle resulted in an outstanding interaction in the prediction of the mean of the 8-category questionnaire SF-36 (*p* < 0.001).

## 4. Discussion

The results of the current study demonstrated an association between FIB-4, a surrogate oxidative marker of liver fibrosis and damage with antioxidant lifestyles and quality of life in patients without apparent liver disease. To our knowledge, this is a pioneer proof of concept description of the association of liver oxidative status and environment impact in a real-life clinical setting.

In the methodological arena, FIB-4 index has demonstrated a linear capacity of hepatic status definition in different cohorts of patients [9,10]. Although the mainstream of evidence in this setting is related to intermediate and final stages of hepatic disease, this score has also served to discriminate the risk of cirrhosis and hepatocarcinoma development with different cut-off values, showing robustness in different pathological scenarios [11,12]. These features also support the plausibility of our study and outcomes. The exploration of oxidative processes in the liver with FIB-4 is also an easy to reproduce process since transaminases and platelet count measurement are widespread and standardized [9].

The inclusion of diseases via the Charlson Comorbidity Index (CCI) to assess disease burden is another strength of the study, since CCI is a validated co-morbidity resume with a wide application, in different clinical settings [30,31]. Furthermore, the collection of data by internal medicine specialists ensures a complete record of patient co-morbidities. The QoL assessment with SF-36 has also been widely validated in diseased patients [32,33,34]. The consecutive recollection of the original ESCAVIDA individuals and the written, on-site self-fulfillment of a validated lifestyle questionnaire may also reinforce the reliability of the study results [35]. 

In this context, the composition of the diet may have an important role in the management of NAFLD [36], since the severity of NAFLD has been associated with an increase in oxidative stress and pro-inflammatory status [37]. Indeed, specific foods such as meat [38], fruits [39], glycemic index [40] or protein content [41] have been involved on pro-oxidative and antioxidant patterns in liver patients. Furthermore, lifestyle factors have been associated with NAFLD in a Mediterranean cohort [42], which have repeatedly reported that physical activity, exercise and sport practice are related to oxidative stress [43,44]. These concepts support the interpretation of our data that oxidative habits are involved in an impaired redox status such as liver fibrosis when assessed with an equation including age, transaminases and platelets [9]. Thus, our findings are in agreement with previous data demonstrating that total dietary pro-oxidant capacity is negatively associated with some metabolic syndrome features [45], as well as in obese subjects [46], where objective markers such as OX-LDL were measured to account for oxidative status. The evaluation of dichotomic specific habits in contrast to the evaluation of a dietary pattern, might allow a more immediate and practical clinical application of the results from this study. The criteria for recording the pro-oxidant dietary habits have been used in highly referenced reports, as part of the Mediterranean dietary pattern, which may reinforce the consistency and reproducibility of the study results. Additionally, although the yes/no rule to detect sedentarism might lack individualization capacity, this strategy could simplify the detection of apparently healthy individuals at risk, depending on lifestyle habits.

The population of the study is a cornerstone in the proof of concept. Thus, FIB-4 scores from the present population are much lower than the cohort of validation of FIB-4 and other cohorts that use this surrogate marker for liver fibrosis. An explanation of this finding is that fibrosis has been looked for and evaluated in specific subgroups in the past [9]. Nevertheless, the single analysis of FIB-4 components show that every component is related to QoL with a plausible trend and that there is an additive improvement of FIB-4 components in the prediction of single QoL SF-36 categories. This evaluation of liver affection in apparently liver disease-free patients contributes to the understanding of the fibrosis scores. Furthermore, FIB-4 discrimination capacity might enhance the affordable screening for NAFLD to apply lifestyle measures as soon as possible. Additionally, the plausibility of the current results, associating oxidative habits related to dietary and physical activity practices in addition to co-morbidity to early stages of fibrosis, supports the idea of the capability of simple scores to be related to actual fibrosis risk under an oxidative perspective.

The addition of QoL categories to FIB-4 prediction provides an interesting scope for fibrosis assessment and also sheds light on a potential therapeutic tool in reducing fibrosis risk by the improvement of quality of life. Although the effect of increasing quality of life in patients with liver fibrosis should be demonstrated in longitudinal studies, this association is a step towards precision medicine and to the empowerment in health of the population, associating a common concept—quality of life—with a complex medical concept such as liver fibrosis [47]. In fact, our results evidence the complex interrelationships between lifestyle, fibrosis, disease and quality of life. The inverse effect of antioxidant lifestyle in quality of life in the morbidity extreme subgroups—not diseased/not pro-fibrotic and diseased/fibrotic patients—reveals an interplay between morbidity and lifestyle in QoL, and should contribute to the development of new strategies for pursuing good habits and improving quality of life in specific subgroups.

This study should be considered a proof of concept, where some limitations should be considered. The cross-sectional design of the study prevents one from assessing causality between the different approaches to liver fibrosis. In addition, the sample size is not as big as other NAFLD/NASH cohorts, which might reduce the statistical power of the study, where type I or type II errors cannot be discarded. Meanwhile, the apparently liver disease-free population selection, the absence of invasive procedures, the non-expensive assessment of fibrosis and the plausibility of the findings, may at least support further, prospectively designed research in this field.

The demonstration of an easy way to determine liver fibrosis, which is associated with quality of life, antioxidant lifestyle and disease burden, provides an interesting tool in the NAFLD scenario. These findings might expand the capacity of evaluation of liver fibrosis in the general medical consultation, making fatty liver disease an evaluable co-morbidity and thus allowing an adequate picture of the NAFLD problem. Additionally, the effect of an antioxidant lifestyle on fibrosis should enhance the investigation not only in beneficial physical activity and dietary patterns but in strategies for convincing the general population of the benefits of a redox balanced way of life. The increasing concern for quality of life in the general population could be a “meeting ground” between medical doctors and patients in the fight against liver and cardiometabolic disease, according to the study results. This synergic effort between physician and patient could carry precision medicine to the next level: individual health empowerment, where the antioxidant/pro-oxidant balance plays a role.

## 5. Conclusions

Liver fibrosis assessed by the FIB-4 index is associated with the interaction between antioxidant lifestyle, co-morbidity and physical, social and general aspects of quality of life in apparent liver disease-free individuals, which opens the door for the prevention and management of NAFLD with a precision medicine scope and a role for patient health empowerment taking into account oxidative issues.

## Figures and Tables

**Table 1:** Population characteristics (*n* = 116).

Variable	Mean (SD) or *n* (%)
Age (years)	58.53 (18.12)
Sex (% female)	59 (50.90)
Morbidity	N of patients with morbidity/Total population
Myocardial infarction, *n* (%)	12 (10.30)
Heart failure, *n* (%)	4 (3.40)
Peripheral vascular disease, *n* (%)	7 (6.00)
Diabetes mellitus, *n* (%)	13 (11.20)
Cerebrovascular disease, *n* (%)	10 (8.60)
Dementia, *n* (%)	5 (4.30)
Chronic obstructive pulmonary disease *n* (%)	9 (7.80)
Connective tissue disease, *n* (%)	13 (11.20)
Renal disease, *n* (%)	6 (5.20)
Hematologic cancer, *n* (%)	1 (0.90)
Solid tumoral disease, *n* (%)	15 (12.90)
Co-morbidities (sum of diseases)	0.93 (1.06)
Frequent medications	
Antihypertensive drugs	38 (32.76%)
Lipid-lowering drugs	21 (18.10%)
Anti-diabetic drugs	6 (5.17%)
Oxidant lifestyle	
Dietary oxidant habits (yes)	57 (49.10)
Absence of exercise (yes)	24 (20.70)
Antioxidative lifestyle *	51 (44.00)
Intermediate lifestyle **	49 (42.20)
Prooxidant lifestyle ***	16 (13.80)
Hepatic liver fibrosis	
Quality of life SF-36 categories (0–100)	
Physical	78.55 (28.60)
Physical role	76.75 (25.38)
Emotional role	86.67 (21.58)
Mental	69.90 (20.19)
Social	78.62 (26.25)
Vitality	55.56 (22.58)
Pain	64.18 (27.12)
General	55.00 (20.19)
Physical summary	63.17 (22.68)
FIB-4 index (points)	0.65 (0.57)

* Neither oxidative dietary nor activity habits, ** Either oxidative dietary or activity habits, *** Both dietary and activity oxidative habits. See Appendix A Table A1.

**Table 2:** Univariable analysis of Fibrosis-4 (FIB-4) according to comorbidity, oxidant lifestyle and quality of life.

Variable	Mean FIB-4 (SD)	*p*
Comorbidity (sum of diseases) *		
No disease	0.51 (0.44)	<0.01
Single disease	0.75 (0.65)	
2 or more diseases	1.01 (0.67)	
Oxidant lifestyle *		
Antioxidant lifestyle *	0.57 (0.39)	0.036
Intermediate lifestyle **	0.77 (0.66)	
Pro-oxidant lifestyle ***	0.99 (0.84)	
Quality of life divided in terciles		
Physical QoL *		
Low	0.90 (0.70)	0.002
Intermediate	0.76 (0.59)	
High	0.44 (0.39)	
Physical role QoL *		
Low	0.89 (0.80)	0.027
Intermediate	0.55 (0.32)	
High	0.61 (0.49)	
Emotional QoL		
Low	0.73 (0.61)	0.317
Intermediate	0.29 (0.13)	
High	0.65 (0.55)	
Mental QoL		
Low	0.83 (0.73)	0.112
Intermediate	0.53 (0.43)	
High	0.68 (0.57)	
Social QoL *		
Low	0.94 (0.84)	0.005
Intermediate	0.47 (0.27)	
High	0.63 (0.42)	
Vitality QoL		
Low	0.69 (0.50)	0.856
Intermediate	0.71 (0.74)	
High	0.64 (0.53)	
Pain QoL		
Low	0.78 (0.70)	0.416
Intermediate	0.60 (0.39)	
High	0.70 (0.63)	
General QoL *		
Low	0.89 (0.74)	0.016
Intermediate	0.64 (0.57)	
High	0.50 (0.34)	
SF-36 Physical Summary QoL *		
Low	0.89 (0.75)	0.012
Intermediate	0.58 (0.48)	
High	0.51 (0.33)	

* No oxidative dietary or activity habits ** Either oxidative dietary or activity habits *** Both dietary and activity oxidative habits. See Appendix A Table A1.

**Table 3:** Multivariable analysis of FIB-4 adjusted to comorbidity, oxidant lifestyle and quality of life.

Physical Quality of Life (QoL)		
Variable	B (SE)	*p*
Sex	0.03 (0.26)	0.813
Morbidity *	0.178 (0.07)	0.014
Oxidative lifestyle **	0.138 (0.076)	0.07
Physical Qol in tertiles	−0.264 (0.119)	0.018
Physical Role QoL		
Variable	B (SE)	*p*
Sex	−0.024 (0.105)	0.817
Morbidity *	0.251 (0.066)	<0.001
Oxidative lifestyle **	0.150 (0.075)	0.049
Physical Role lowest tercile	0.259 (0.116)	0.027
Social QoL		
Variable	B (SE)	*p*
Sex	−0.16 (0.104)	0.876
Morbidity *	0.236 (0.065)	<0.001
Oxidative lifestyle **	0.166 (0.073)	0.026
Social Role lowest tercile	0.315 (0.111)	0.005
General QoL		
Variable	B (SE)	*p*
Sex	−0.12 (0.106)	0.909
No morbidity ***	−0.337 (0.108)	0.002
Oxidative lifestyle **	0.157 (0.077)	0.043
General QoL lowest tercile	0.229 (0.113)	0.046

* Morbidity in 3 subgroups (0, one or more diseases in the CCI index) ** Oxidative lifestyle in 3 subgroups (zero, one or two points according to the previously described definition of pro-oxidant lifestyle, Appendix A Table A1) *** No morbidity in 2 subgroups (No/Yes).

## Appendix A

**Table A1:** Pro-oxidant lifestyle definitions.

Pro-oxidant Habits	Pro-oxidant Lifestyle Calculation		Oxidative Lifestyle	
Ultra-processed pastries consumption ≥ 3 times/week	Pastries or carbonated beverages consumption	+1 point	Antioxidant lifestyle	0 point
Carbonated beverages consumption ≥ 1/day	Absence of physical activity	+1 point	Intermediate lifestyle	1 point
Absence of regular physical activity	Range	0–2 points	Pro-oxidant lifestyle	2 points

**Table A2:** Association between components of Fibrosis-4 (FIB-4) and the 8 categories of quality of life (QoL) in SF-36.

QoL Category	Age	GOT/sqrGPT	Platelet Count	GOT/sqrGPT * Platelets	FIB-4
Physical	−0.447 * (0.001)	−0.083 (0.408)	0.157 (0.118)	−0.233 * (0.019)	−0.406 * (0.001)
Physical role	−0.170 (0.090)	−0.018 (0.855)	0.275 * (0.005)	−0.216 * (0.030)	−0.299 * (0.002)
Emotional role	−0.141 (0.160)	−0.034 (0.738)	0.075 (0.456)	−0.058 (0.562)	−0.137 (0.173)
Mental	−0.103 (0.306)	−0.080 (0.426)	0.091 (0.367)	−0.125 (0.213)	−0.145 (0.149)
Social	−0.120 (0.231)	−0.066 (0.511)	0.157 (0.118)	−0.209 * (0.036)	−0.286 * (0.004)
Vitality	−0.053 (0.595)	0.057 (0.574)	0.109 (0.278)	−0.048 (0.634)	−0.060 (0.549)
Pain	−0.007 (0.942)	−0.147 (0.141)	0.094 (0.351)	−0.220 * (0.027)	−0.175 (0.080)
General	−0.170 (0.089)	−0.138 (0.168)	0.256 * (0.010)	−0.259 * (0.009)	−0.284 * (0.004)

sqrGPT: Square root of GPT. Results are Pearson r (*p* value). * Statistically significant results.
